# Relative importance of meteorological and geographical factors in the distribution of *Fasciola hepatica* infestation in farmed sheep in Qinghai province, China

**DOI:** 10.1051/parasite/2016070

**Published:** 2016-12-21

**Authors:** Hongyu Qin, Xiang Gao, Hongbin Wang, Jianhua Xiao

**Affiliations:** 1 Department of Veterinary Surgery, Northeast Agricultural University Harbin Heilongjiang 150030 PR China

**Keywords:** *Fasciola hepatica*, Parasite, Meteorological and geographical factors, Risk analysis, Prediction map

## Abstract

*Fasciola hepatica* is an important trematode parasite of economic importance that infests sheep and cattle worldwide. We conducted a detailed investigation into the spatial distribution of *F. hepatica* infestation in farmed sheep in Qinghai (Wutumeiren) province, Mainland China. Mathematical modelling was used to assess the inter-relationships between meteorological and geographical factors and the risk of *F. hepatica* infestation across the province. A capture enzyme-linked immunosorbent assay (ELISA) test (MM3-SERO) was used to detect *F. hepatica* infestation. A niche model based on the maximum entropy method (MaxEnt) was used to estimate the influence of meteorological and geographical factors on the observed spatial distribution of *F. hepatica* infestation. Results of jackknife analysis indicated that temperature, precipitation, solar radiation, digital elevation and slope were associated with the occurrence of *F. hepatica* infestation, and that infestation rates were significantly higher among animals from districts with a high percentage of grassland habitat. The findings indicate that meteorological and geographical factors may be important variables affecting the distribution of *F. hepatica* infestation and should be taken into account in the development of future surveillance and control programmes for fascioliasis.

## Abstract (fr)

*Fasciola hepatica* est un important trématode parasite, d’importance économique, qui infeste les moutons et les bovins dans le monde entier. Nous avons mené une enquête détaillée sur la répartition spatiale de l’infestation par *F. hepatica* chez des moutons d’élevage dans la province de Qinghai (Wutumeiren), en Chine continentale. La modélisation mathématique a été utilisée pour évaluer les interrelations entre les facteurs météorologiques et géographiques et le risque d’infestation par *F. hepatica* dans toute la province. Un test ELISA de capture (MM3-SERO) a été utilisé pour détecter l’infestation par *F. hepatica*. Un modèle de niche basé sur la méthode de l’entropie maximale (Maxent) a été utilisé pour estimer l’influence des facteurs météorologiques et géographiques sur la répartition spatiale observée de l’infestation par *F. hepatica*. Les résultats de l’analyse de Jackknife ont indiqué que la température, les précipitations, le rayonnement solaire, l’élévation numérique et la pente étaient associés à l’apparition d’une infestation par *F. hepatica* et que les taux d’infestation étaient significativement plus élevés chez les animaux provenant de districts où l’habitat des prairies était élevé. Les résultats indiquent que les facteurs météorologiques et géographiques peuvent être des variables importantes affectant la distribution de l’infestation par *F. hepatica* et devraient être pris en compte dans l’élaboration de futurs programmes de surveillance et de lutte contre la fasciolose.

## Introduction

*Fasciola hepatica* (*F. hepatica*), also known as the common liver fluke, is a parasite that is widespread throughout the world [13]. The species utilizes snails of the Lymnaeidae family as its intermediate host [15]. Infestation in the primary host, commonly livestock of various species, causes fascioliasis, a disease which has a major economic impact on livestock productivity due to its negative effects on milk yield, meat production and fertility, as well as high mortality rates. It is estimated that fascioliasis infestations in cattle result in annual losses of €30 per cow across the dairy population in Flanders, Belgium, and €299 per infested cow in Switzerland [3, 30]. Moreover, farmed animal species such as sheep and cattle may play an important role as reservoirs for human fascioliasis infestation in endemic areas [15].

In recent years, fascioliasis has emerged or re-emerged in many countries, both in animal and human populations [14]. This may be due to changes in local climate conditions. Previous studies have indicated that both *F. hepatica* and its snail host can respond rapidly to variations in environmental conditions [21, 29]. Small increases in temperature and rainfall can extend the window of contamination risk leading to higher transmission and infestation rates [14, 34]. Because Geographic Information Systems (GIS) can rapidly and directly associate climatic and topographical variables with species presence/absence data [1, 24], they can be used to make predictions about the distribution of species. On this basis, geographical spatial risk models of fascioliasis have been developed in many countries [6, 17, 34, 35].

In this study, spatial risk analysis was performed by means of the maximum entropy method (MaxEnt) [25]. MaxEnt can generate inferences from limited information about the species presence and has shown high prediction accuracy [18]. Furthermore, it can evaluate the contribution of each environmental factor to the distribution of the species and ultimately produce a predictive distribution map. Thus, it has been extensively used for predicting the potential distributions of plants, animals and insects [5, 26, 33].

The aim of this study was to assess the influence of meteorological and geographical factors on the distribution of *F. hepatica* infestation in sheep, by using the MaxEnt model and data collected from the Qinghai province of mainland China. It is anticipated that the findings will inform the current understanding of the epidemiology of *F. hepatica* and provide information relevant to the design of more effective control strategies for the eradication of fascioliasis.

## Materials and methods

### Study area

Qinghai province is located in the north of Mainland China. It extends between latitude 31°9′ and 39°19′ N and longitude 89°35′ and 103°04′ E. Qinghai province has a plateau continental climate. The temperature and rainfall have marked seasonal variation: average temperatures range from −5.0 °C to −10.3 °C in winter and 10.8 °C to 19.0 °C in summer. The majority of the rainfall occurs in the wet season, from June to October. The province has a long dry season from November to May, coinciding with the lowest minimum temperatures. Because of the high altitude of the region, the level of solar radiation is high. Total annual solar radiation can be up to 690,800–753,600 joules per square centimeter. Mountains are mainly distributed in the western parts of the province, and the Qaidam Basin occupies the eastern part of the province. The basin is the main pastoral area because it consists of a large number of lakes and swamps. The grassland area of the province is 40.34 million hectares, which represents 12% of the total grassland area of Mainland China.

### Testing of sheep herds for *F. hepatica* infestation

Every year from 2012 to 2015, all sheep herds in Qinghai province were tested for *F. hepatica* infestation. Blood samples were collected from sheep > 6 months of age in every herd, exposure levels were calculated by using percent positivity (PP) values [17]. Herds with a PP value of less than 27 were considered negative for *F. hepatica* infestation. Finally, 84 herds were considered positive for *F. hepatica* infestation in the 4 years. Their geographic coordinates were recorded by using portable GPS (Global Position System) and saved as data points about the infestation presence.

*F. hepatica* testing was carried out as follows: Blood was collected from sheep on the farm and immediately placed in a cooler. Samples were centrifuged within 12 h of collection to obtain sera. Serum samples were stored frozen at −20 °C and sent within 3 days to the Biology Laboratory of Qinghai University, China, for analysis. Detection of *F. hepatica* was based upon the MM3-SERO capture ELISA test [22]. Firstly, serum samples were diluted to 1% by addition of 1% skimmed milk powder and phosphate-buffered saline (PBS) containing 0.2% Tween-20 (PBS-T). Subsequently, diluted samples were added to 96-well ELISA plates containing either MM3 alone or MM3-Ag complexes and incubated at 37 °C for 2 h. A peroxidase-conjugated monoclonal antibody to sheep IgG (Sigma-Aldrich) that had been diluted 1:30,000 was added to each well and incubated for 90 min at 37 °C. O-phenylenediamine (SigmaFast OPD tablets, Sigma-Aldrich) was then added and the incubation continued for 20 min at room temperature. Finally, 25 μL of 3 M H_2_SO_4_ was used to stop the reaction and the optical density (OD) was measured at 492 nm using a spectrophotometer. The cut-off value was calculated on the basis of OD values obtained from the sera of sheep that had not been exposed to *F. hepatica* infestation.

### Meteorological and geographical variables

Meteorological data for the years 2009–2015 were included in the analysis and obtained from the China Meteorological Data Sharing System (http://data.cma.cn/) [37]. This network provides a high spatial resolution of approximately 1 km^2^ for gridded climate data across China and has been frequently used for spatial ecological modelling and mapping [8]. In the present analysis, individual years were defined as the period between 1 November of the previous year and 31 October of the present year.

The geographical data came from the “Land Cover Classification Database” of the University of Maryland, United States (http://www.landcover.org/data/landcover/). These data had been produced from satellite data obtained from the Advanced Very High Resolution Radiometer (AVHRR), a cross-track scanning system with five spectral bands having a resolution of 1.1 km and a frequency of earth scans twice per day. In addition, digital elevation and slope data were included in the analysis. These data were obtained from the National Geomatics Center of China (http://ngcc.sbsm.gov.cn/). The meteorological, elevation and geographical data were all inputted into a raster map by means of ArcGIS 10.2 software (ESRI, Redlands, CA, USA). Table 1 shows the risk variables inputted into the model.

**Table 1.** Risk variables inputted into the model.

Type of variable	Name of variable	Abbreviation
Land cover	Water	L1
	Deciduous Needleleaf Forest	L2
	Deciduous Broadleaf Forest	L3
	Theropencedrymion	L4
	Wooded Grassland	L5
	Grassland	L6
	Closed Shrubland	L7
	Open Shrubland	L8
	Cropland	L9
	Bare Ground	L10
	Urban and Built	L11
	Slope	L12
	Digital elevation	L13
Meteorological variables	Monthly maximum temperature	M1
	Monthly minimum temperature	M2
	Mean monthly temperature	M3
	Monthly temperature range	M4
	Relative humidity	M5
	Rainfall	M6
	Solar radiation	M7

### Predictive modelling

The MaxEnt model was used to analyse the effect of meteorological and geographical factors on the spatial distribution of *F. hepatica* infestation in sheep in Qinghai province. MaxEnt can make predictions of species distribution based on incomplete data and can estimate the relative importance of each identified risk variable by means of the jackknife test [9]. To develop and test the model produced by this analysis, 75% of the data were used as training data and the remaining 25% were used to test the model’s predictive ability [36].

Evaluation of the model’s performance in predicting the distribution of *F. hepatica* infestation was based on the Area Under the Curve (AUC) of the Receiver Operating Characteristic Curve (ROC), as developed by Peterson et al. [23]. AUC may range from 0.5 to 1 and higher AUC indicates better model performance. Sensitivity, which is also named the true positive rate, can measure the ability to correctly identify diseased sheep. Its value equals the rate of true positive and the sum value of true positive and false negative. Specificity, which is also named the true negative rate, can measure the ability to correctly identify non-diseased sheep. Its value equals the rate of true negative and the sum value of false positive and true negative. The value of the AUC was calculated by R-statistical analysis software, version 2.15.1 [32].

## Results

### Assessment of the accuracy of the predictive model

**Figure 1.**
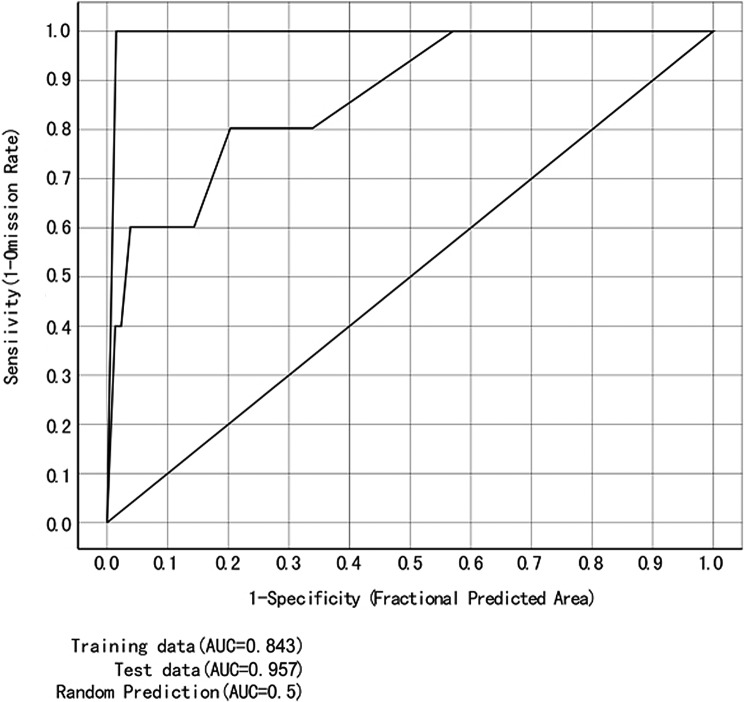
ROC curves produced by the MaxEnt model. AUC values of greater than 0.8 indicate high performance (high predictive ability) of the model.

A higher AUC indicates better performance and stronger predictive power of the MaxEnt model. The final values of the AUC in the present study were 0.843 and 0.957, for the training and testing data sets, respectively. This indicates that the predictions made by the MaxEnt model were relatively accurate (Fig. 1).

### Relative importance of the variables

**Figure 2.**
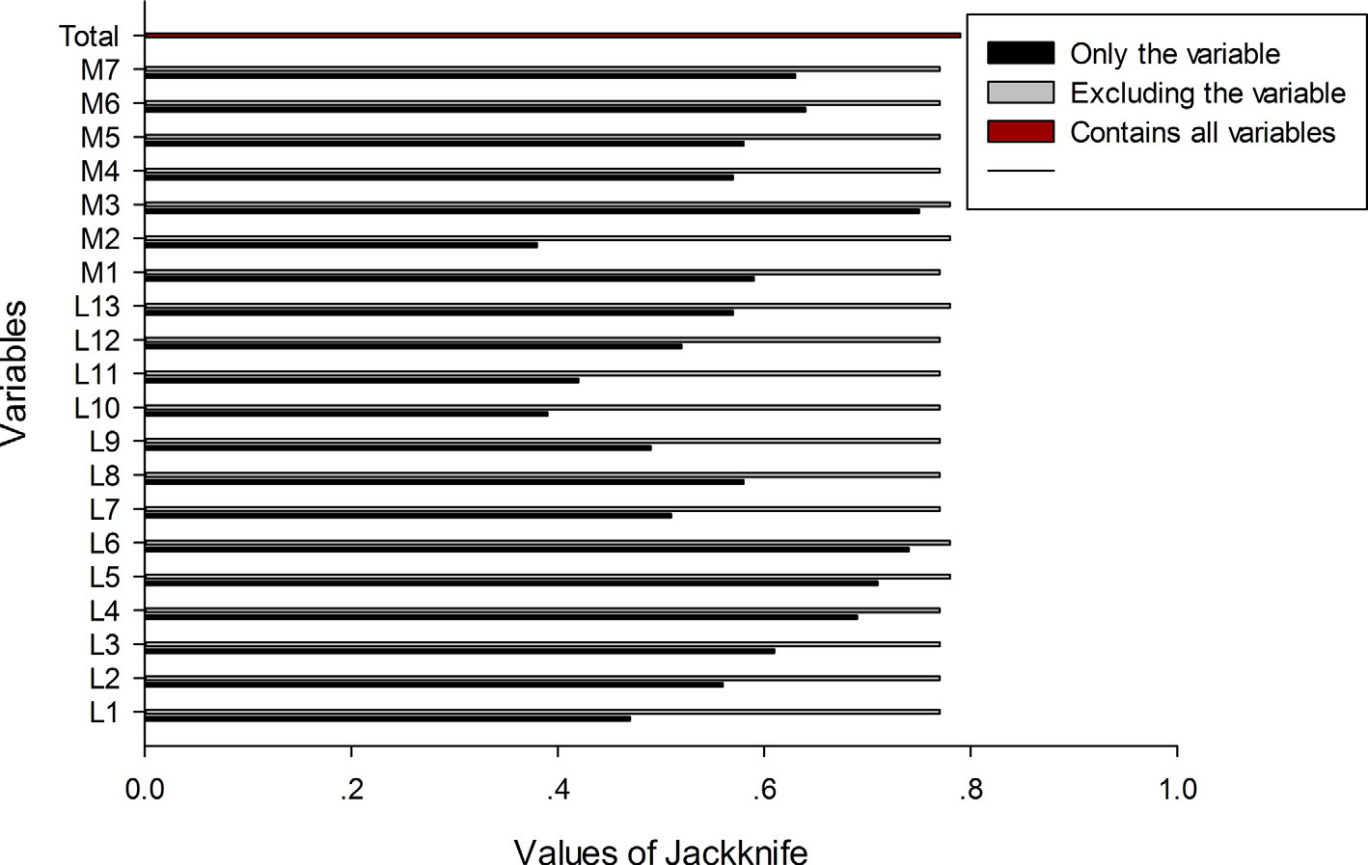
Relative importance of meteorological variables in predicting the probability of *F. hepatica* infestation, as determined by the jackknife analysis. Variables producing higher trainings gain are considered to be more important (more predictive). L1 – Water; L2 – Deciduous Needleleaf Forest; L3 – Deciduous Broadleaf Forest; L4 – Theropencedrymion; L5 – Wooded Grassland; L6 – Grassland; L7 – Closed Shrubland; L8 – Open Shrubland; L9 – Cropland; L10 – Bare Ground; L11 – Urban and Built; L12 – Slope; L13 – Digital elevation; M1 – Monthly maximum temperature; M2 – Monthly minimum temperature; M3 – Mean monthly temperature; M4 – Monthly temperature range; M5 – Relative humidity; M6 – Rainfall; M7 – Solar radiation.

Finally, 84 herds were considered positive for *F. hepatica* infestation in our survey. These 84 herds mainly distributed in six prefectures of Qinghai province (Table 2). Figure 2 displays the results of the jackknife analysis. These results show that, among the meteorological variables included in the model, mean monthly temperature, precipitation, solar radiation, digital elevation and slope had the most significant effect on the probability of *F. hepatica* infestation, and that *F. hepatica* infestation occurred most frequently in the regions with the highest percentage of grassland.

**Table 2.** The number of *F. hepatica* infestation positive sheep herds in Qinghai Province.

Prefecture	Total	2012	2013	2014	2015
Minhe	10	1	3	4	2
Hualong	7	1	2	3	1
Wulan	16	2	5	5	4
Gonghe	18	3	7	6	2
Huzhu	12	3	3	5	1
Gangcha	13	3	5	3	2
Nangqian	3	0	2	1	0

*Note*: Five species distributed in the Hoh Xil, which is a nature reserve and does not belong to any prefecture.

### Prediction map of *F. hepatica* distribution

**Figure 3.**
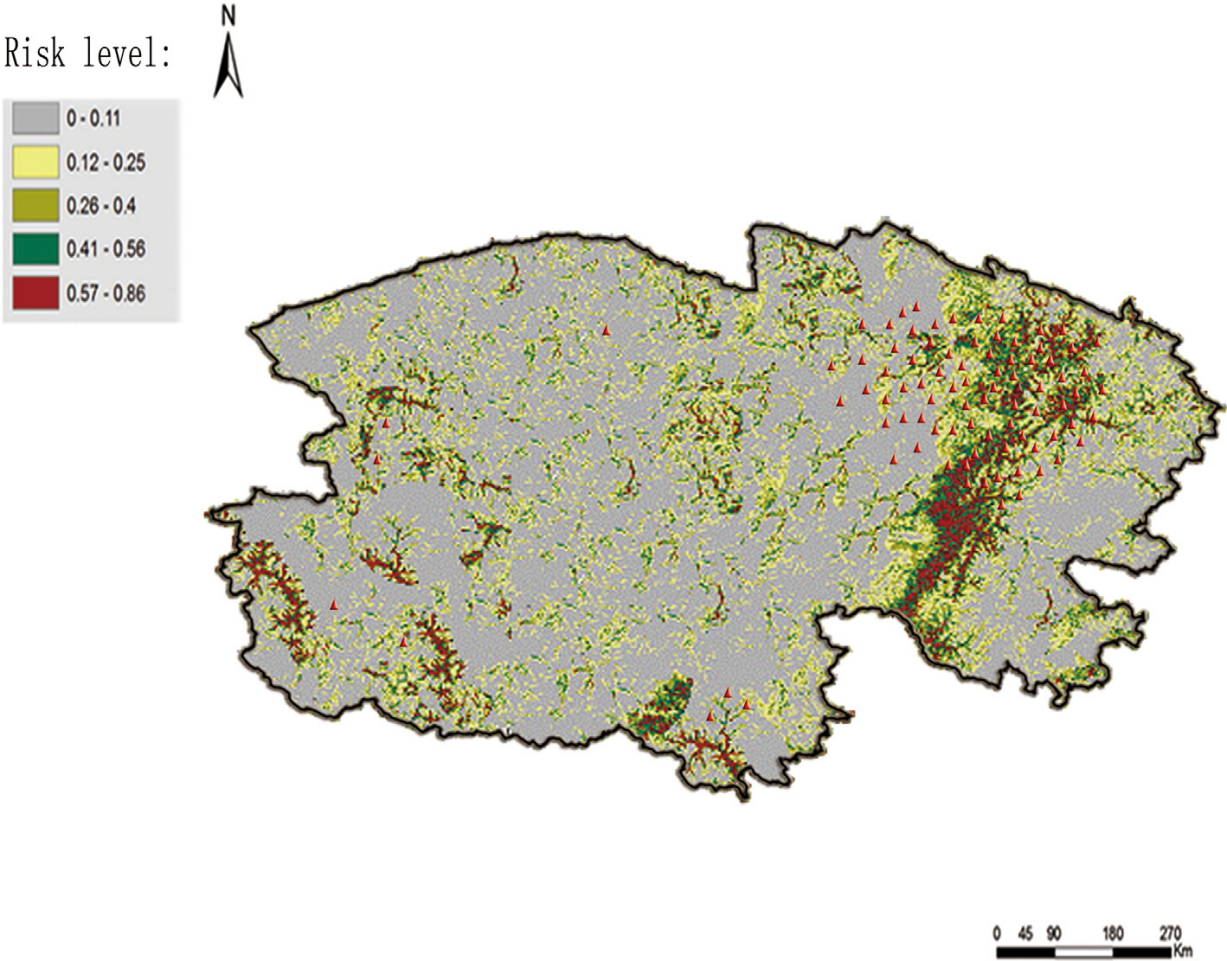
Risk map of *F. hepatica* infestation predicted by MaxEnt. The highest risk of infestation with *F. hepatica* is situated in the east of Qinghai province. The red triangles represent the location of sheep herds considered positive for *F. hepatica* infestation.

Figure 3 displays the potential geographic distribution of *F. hepatica* infestation as predicted by the MaxEnt program. According to the predicted distribution, the eastern part of Qinghai province carries the highest risk level for *F. hepatica* infestation, extending to the southern and south-western regions of the province. The Qaidam Basin and Qinghai Lake are located in the east of Qinghai province, thus, this region has more dense vegetation. Compared with the north-western and central regions, which are mainly mountainous land, the southern and south-western regions of Qinghai province also have lower altitude and a warmer and more humid climate.

## Discussion

The ability of climatic conditions to modulate the extent and intensity of *F. hepatica* infestations is well known [14, 21]. In the present study, the results of the jackknife analysis revealed that temperature, precipitation, solar radiation, digital elevation, slope and grassland may be among the important variables influencing the occurrence of *F. hepatica* infestations in sheep.

Weather conditions can affect the incidence of both *F. hepatica* itself and its intermediate hosts [14, 20]. In the case of *F. hepatica*, temperature can have a pronounced influence on cercariae production in the intermediate host, which is a crucial step in the life cycle of trematode worms [7]. Kendall and McCullough [10] demonstrated that *F. hepatica* ceases development and its cercariae are not liberated from the snail intermediate host when environmental temperatures fall below 10 °C. An analysis by Poulin [27] similarly reported that the rate of cercarial emergence from snail hosts is markedly higher when temperatures are around 20 °C, compared with lower temperatures. This study also indicated that the snail’s physiological growth rate, which reflects enzymatic reactions in the snail’s body, is doubled or tripled with every 10 °C increase in temperature.

Rainfall and moisture are necessary for the development of *F. hepatica*. At the same time, rainfall has a positive effect on the survival and reproduction of the snail intermediate host [19]. At the right level, rainfall can create humid microhabitats which benefit *F. hepatica*, whilst on the other hand, large amounts of rainfall will inhibit *F. hepatica* infestation because it can wash away the parasite larvae separating them from the snail hosts [12, 28]. In a study by Rapsch [34], 210 mm of rainfall was shown to be a threshold level in this respect. When precipitation goes beyond this value, the risk of *F. hepatica* infestation decreases rapidly. In Qinghai province, annual precipitation ranges from 100 to 180 mm. It would therefore appear that rainfall in this region provides sufficient humidity for the survival of *F. hepatica* and its intermediate host snails.

Solar radiation also showed a positive correlation with the occurrence of *F. hepatica* infestation in the present study. This may be because algae are the major food source of the snail hosts [34] and a high level of solar radiation has a positive effect on the growth rates of algae.

Previous studies have indicated that land supporting dense vegetation carries a high risk of *F. hepatica* infestation [34]. Because soils with dense vegetation, such as grassland, woodland and forest, inherently have low drainage and high water retentive capacities, they tend to support smaller water bodies and water courses which are the preferred habitat of the Lymnaeidae [2, 4, 11]. Compared with grassland, forest and woodland have been shown to confer a lower risk of fascioliasis infestation because of the lower level of solar radiation in these habitat types. Moreover, grassland is typically used for grazing and is thus more likely to be contaminated with the faeces of infested livestock containing *F. hepatica* eggs [20]. Therefore, grassland with low water permeability and high water retention properties confers a higher risk of *F. hepatica* infestation than other soil types.

According to the prediction maps generated by the present study, the most probable distribution areas for *F. hepatica* were located in the East of Qinghai province. This may reflect the land use, elevation and slope of the eastern region, which could affect the survival of *F. hepatica* and its intermediate host. The Qaidam Basin occupies the east of the province and has a high concentration of grassland which is a habitat known to have a higher risk of infestation with *F. hepatica*, as discussed above. In addition, because of the low altitudes in the eastern area, average temperatures tend to be higher than in other regions of the province. Warm temperatures in the east may have promoted prolonged survival of *F. hepatica* and its snail hosts [14]. Finally, compared with the hilly lands in the western regions of the province, the lowland eastern areas have a low level of digital elevation, and slope data and drainage in these areas are impeded. Rainfall always exceeds potential transpiration in these areas and thus is beneficial for the luxuriant vegetation and the conservation of smaller water bodies and water courses in these areas [21]. Thus, the environment of the eastern regions of Qinghai province is more suitable for the survival of *F. hepatica* and its intermediate host freshwater snails.

MaxEnt is well known as a presence-only ecological niche model [16]. It can make predictions about species distribution based on limited data and has shown high prediction accuracy [18]. The previously mentioned study by Stockwell [31] indicated that the MaxEnt model can make predictions with a high level of accuracy when only 50 data points were included. In comparison with these studies, the present study included 84 data points.

In addition to the environmental variables considered in this study, other variables such as those linked with human intervention, the presence of snail-suitable water vegetation, groundwater quality and socio-economic factors may also be relevant influencing variables on the distribution of *F. hepatica* infestation, and were not assessed in the present study. In future work, additional data relating to these factors will be collected and the predictive model expanded in order to improve the accuracy of the predictions.

## Conclusion

The results of this study indicate that meteorological and geographical factors can affect the distribution of *F. hepatica* infestation in sheep in the Qinghai province of Mainland China. Based upon this knowledge, an approach can be applied to design better and more evidence-based planning and detailed surveillance programmes for fascioliasis, involving more efficient use of the limited funding and human resources that are available. The current control strategies used for fascioliasis in Qinghai include using anthelminthics (niclofolan, albendazole, triclabendazole and ivermectin) to expel the parasitic worms from the infested animals, and molluscicides for the elimination of the intermediate snail hosts. Based on the findings of the present study, herdsmen in Qinghai province could enhance the positive effects of these drugs by more timely application, based upon knowledge of which meteorological factors influence infestation rates. Furthermore, the predictions of the model we generated could also help to guide herdsmen towards avoiding the habitat “danger zones” of *F. hepatica* infestation during the grazing season and to take measures (such as digging drains and transforming swamps) to destroy the living conditions of the intermediate host. It is hoped that the findings will also inform scientific understanding of the risks of *F. hepatica* infestation spreading from one area to another. Further research, involving the collection of more detailed information for a greater number of potentially influential variables, will further improve the ability to predict changes in *F. hepatica* prevalence and distribution in this region of Mainland China.
